# Dual roles of anesthetics in postoperative cognitive dysfunction: Regulation of microglial activation through inflammatory signaling pathways

**DOI:** 10.3389/fimmu.2023.1102312

**Published:** 2023-01-27

**Authors:** Mengxue Zhang, Yiqing Yin

**Affiliations:** ^1^ Department of Anesthesiology, Tianjin Medical University Cancer Institute and Hospital, National Clinical Research Center for Cancer, Tianjin, China; ^2^ Tianjin’s Clinical Research Center for Cancer, Tianjin, China; ^3^ Key Laboratory of Cancer Prevention and Therapy, Tianjin, China

**Keywords:** anesthetics, postoperative cognitive dysfunction, microglia, inflammatory signaling pathways, neuroinflammation

## Abstract

Postoperative cognitive dysfunction (POCD) is a prevalent clinical entity following surgery and is characterized by declined neurocognitive function. Neuroinflammation mediated by microglia is the essential mechanism of POCD. Anesthetics are thought to be a major contributor to the development of POCD, as they promote microglial activation and induce neuroinflammation. However, this claim remains controversial. Anesthetics can exert both anti- and pro-inflammatory effects by modulating microglial activation, suggesting that anesthetics may play dual roles in the pathogenesis of POCD. Here, we review the mechanisms by which the commonly used anesthetics regulate microglial activation *via* inflammatory signaling pathways, showing both anti- and pro-inflammatory properties of anesthetics, and indicating how perioperative administration of anesthetics might either relieve or worsen POCD development. The potential for anesthetics to enhance cognitive performance based on their anti-inflammatory properties is further discussed, emphasizing that the beneficial effects of anesthetics vary depending on dose, exposure time, and patients’ characteristics. To minimize the incidence of POCD, we recommend considering these factors to select appropriate anesthetics.

## 1 Introduction

POCD is a common postoperative complication characterized by personality changes and impaired learning and memory capacities (1, 2). The incidence of POCD in elderly surgical patients can reach 41.4%, and it increases postoperative complications and mortality rates (3). The pathogenesis of POCD is still unknown. In recent years, it has become clear that neuroinflammation mediated by microglia plays a key role in the pathogenesis of POCD (4, 5). In response to inflammatory stimuli, microglia, which serve as the first line of defense in the central nervous system (CNS), are activated and polarized into two opposing phenotypes: pro-inflammatory M1 and anti-inflammatory M2 (6, 7). M1 and M2 phenotypes are responsible for the release of pro- and anti-inflammatory mediators, respectively (8). Excessive microglial activation and a dysregulated M1/M2 ratio exacerbate neuroinflammation and impair neurocognitive function (6, 9). Inhibition of microglial activation and promotion of microglial M2 polarization are potential treatment strategies for neuroinflammatory diseases. Therefore, microglia are essential research targets for the pathogenesis of POCD.

The administration of anesthetics is a critical risk factor for POCD, which is associated with the microglial activation and neuroinflammation induced by anesthetics (2, 10, 11). However, recent evidence suggests that anesthetics have both anti- and pro-inflammatory properties and may play dual roles in the pathogenesis of POCD (12–14). Several commonly used anesthetics can improve neurocognitive outcomes by suppressing microglial activation, promoting M2 polarization, and exerting anti-neuroinflammatory effects (15–17). These findings suggest a role for anesthetics in perioperative neuroprotection studies. In contrast, high doses, long-term exposure, and the vulnerable phases of newborns and elderly patients are likely to drive anesthetics to switch from inhibiting microglial activation to promoting it, which increases the risk of anesthetic-induced POCD (18, 19).

Although anesthetics play a key role in the development of POCD, the contribution of anesthesia versus surgery in POCD is difficult to distinguish. In this review, to better understand the anti- and pro-inflammatory mechanisms of anesthetics, we review literature on the independent effect of anesthetics, and introduce intravenous, volatile, and local anesthetics that regulate microglial activation and M1/M2 polarization *via* multiple inflammatory signaling pathways. In particular, we list the anti-inflammatory and neuroprotective effects of anesthetics in various inflammatory models, such as lipopolysaccharide (LPS) stimulation, cerebral ischemia/reperfusion (I/R) injury, and laparotomy surgery. We discuss the potential role of anesthetics in ameliorating POCD by suppressing microglial activation, a topic requiring further exploration. And we suggest anesthesiologists should consider the anti- and pro-inflammatory properties of anesthetics, as well as their dose, exposure time, and patients’ specific characteristics, to minimize the incidence of POCD.

## 2 Microglial activation

Microglia are resident immune cells in the CNS, accounting for 10-15% of all brain cells (20). They are typically in a resting state and secrete neurotrophic factors such as nerve growth factor (NGF) and brain-derived neurotrophic factor (BDNF), which are involved in neuronal development, maintenance, and survival (21). Microglial functions such as synapse pruning and synaptic stripping are essential for regulating synaptic plasticity and maintaining proper learning and memory capabilities (22). In addition, during the development of neuroinflammation processes with the disruption of the blood-brain barrier (BBB) and the infiltration of peripheral immune cells, microglia are activated by several inflammatory mediators (23, 24).

Depending on the activated state, microglial activation can have both neuroprotective and neurotoxic effects. Microglial activation is traditionally classified into two major phenotypes: pro-inflammatory M1 (classical activation) and anti-inflammatory M2 (alternative activation) (8). The M1 phenotype is activated by pro-inflammatory mediators such as tumor necrosis factor-alpha (TNF-α), LPS, and interferon-gamma (IFN-γ) (25), while the M2 phenotype is activated by anti-inflammatory cytokines such as interleukin (IL) -4 and IL-13 (26). Microglial M1 phenotype releases proinflammatory cytokines (such as IL-1β, TNF-α, and IL-6), chemokines, nitric oxide (NO), and reactive oxygen species (ROS), resulting in neuronal cell injury and BBB disruption (27). The neuronal damage mediated by chronic M1 phenotype activation is a component in the pathogenesis of Alzheimer’s disease (AD), Parkinson’s disease (PD), and amyotrophic lateral sclerosis (ALS) (6). The microglial M2 phenotype releases anti-inflammatory cytokines IL-10, arginase (Arg-1) and chitinase-3 (Chil3) to maintain and repair neural tissue (28). The polarization of M2 microglia is essential for the restoration of tissue homeostasis after inflammatory injury (28). Promoting the polarization of microglia from M1 to M2 ameliorates the progression of several neuroinflammatory diseases (6, 29), suggesting a prospective therapeutic potential. Indeed, this dichotomous classification simplifies microglial activation, and multiple intermediate phenotypes between M1 and M2 phenotypes have been identified in recent years (30). However, the regulation of M1/M2 polarization remains a focus in the study of neuroinflammatory disease pathogenesis, and further studies are required.

## 3 Inflammatory signaling pathways involved in microglial activation

Multiple receptors expressed in microglia recognize inflammatory mediators and transmit the inflammatory stimulus signal to induce microglial activation *via* downstream signaling pathways (7, 31), mediating the release of pro-inflammatory cytokines, chemokines, and promoting increased NO and ROS production from microglia, thus contributing to the development of the central neuroinflammatory response (32). Targeting the upstream receptors or the downstream pathways has been a crucial strategy for regulating microglial activation and has promising research prospects for the treatment of neuroinflammatory diseases (31, 33). Therefore, it is vital to recognize the inflammatory signaling pathways involved in microglial activation.

### 3.1 Microglial receptors for inflammatory signal transmission

In addition to chemokine and IL receptors, there are critical components that recognize danger-associated molecular patterns (DAMPs), ligands produced by damaged cells (4). The toll-like receptor (TLR) family of pattern recognition receptors (PRRs) plays a crucial function in recognizing DAMPs (34). Toll-like receptor 4 (TLR4), a member of the TLR family, is overexpressed in microglia in response to inflammatory stimuli (35). TLR4 identifies DAMPs and transmits signals downstream by binding to the cytosolic adaptor protein myeloid differentiation primary response 88 (MyD88) (35, 36). As the upstream signal transduction node, TLR4 mediates the activation of multiple inflammatory signaling pathways, such as the nuclear factor kappa B (NF-κB) pathway, phosphatidylinositol 3-kinase (PI3K)/protein kinase B (Akt) pathway, and mitogen-activated protein kinases (MAPKs) pathway, suggesting its pivotal role in neuroinflammation (37–39). Another surface receptor that identifies DAMPs and mediates the NF-κB pathway activation is the receptor for advanced glycation end products (RAGE) (40), a multiligand receptor involved in non-PRRs (41). Recently, the activation of triggering receptors expressed on myeloid cells 2 (TREM2), a surface receptor expressed on microglia (42), has been shown to exert anti-neuroinflammatory effects *via* the PI3K/Akt pathways (43, 44). These microglial receptors, as nodes of signal transduction, are essential targets for neuroinflammatory mechanisms and may play a significant role in the amelioration of neuroinflammatory diseases.

### 3.2 NF-κB signaling pathway

The transcription factor NF-κB is a key regulator involved in microglial M1 activation (20), based on the role of promoting the expression of numerous inflammatory mediators (45), such as proinflammatory cytokines IL-1, IL-6 and TNF-α, proinflammatory enzymes cyclooxygenase 2 (COX2) and inducible nitric oxide synthase (iNOS) (45). NF-κB exists in multiple dimeric forms, mostly as RelA (p65)/P50 complexes that are involved in the canonical NF-kB pathways activated by proinflammatory cytokines, LPS, and DAMPs (46, 47). As a member of DAMPs, high mobility group box 1 (HMGB1) has been identified as a major neuroinflammatory biomarker associated with cognitive impairments (48, 49). HMGB1 is recognized by both TLR4 and RAGE on microglia (35, 40), and the inactive NF-κB p65/p50 in cytosolic is released from the NF-κB inhibitor IκB, where it becomes active to enter the nucleus, binding to the promoter region of pro-inflammatory genes and promoting the expression of proinflammatory mediators (47). Studies focusing on the inhibition of the HMGB1/TLR4/NF-κB and HMGB1/RAGE/NF-κB axes showed a suppression of microglial M1 polarization and promotion of M2 polarization, resulting in neuroprotective benefits (40, 50).

### 3.3 PI3K/AKT signaling pathway

PI3K is an intracellular lipid kinase that transduces signals from microglial surface receptors such as TLR4 and tyrosine receptor kinase B (TrkB) and activates Akt *via* phosphorylation of phosphatidylinositol 4,5 bisphosphate (PI (4, 5)P2) to phosphatidylinositol 3,4,5 trisphosphate (PI (3–5)P3) (51, 52). Phosphorylated Akt can activate NF-κB and mediate inflammation; inhibiting the microglial PI3K/Akt/NF-κB pathway reduces microglial activation and the release of pro-inflammatory cytokines (53). Glycogen synthase kinase-3 beta (GSK-3β), as a serine/threonine kinase, can be inactivated by phosphorylated Akt to activate nuclear factor erythroid 2-related factor 2 (Nrf2) (54), thus facilitating the microglial M2 polarization (55). In addition, new evidence suggests that microglial TREM2 activation reduces microglia-mediated neuroinflammation and ameliorates cognitive impairment *via* the PI3K/Akt signaling pathway (43, 44), indicating the TREM2/PI3K/Akt pathway may be a potential neuroprotective target. Together, the PI3K/Akt pathway is involved in microglial activation and M1/M2 phenotype polarization, exerting both anti- and pro-inflammatory effects.

### 3.4 MAPK signaling pathway

The MAPKs family, as intracellular signaling molecules, are comprised of extracellular signal-regulated kinase (ERK), p38, and c-Jun N-terminal kinase (JNK) and regulating inflammatory responses (56). NF-κB is also the MAPK family’s downstream activated molecule (57, 58). The activation of microglial MAPKs pathways enhanced the release of pro-inflammatory cytokines and promoted the microglial M1 polarization through the activated NF-κB (58). *In vivo* and *in vitro* studies revealed that inhibiting the MAPKs/NF-κB pathway can attenuate microglia-mediated neuroinflammation (59, 60). Furthermore, inhibiting the MAPKs/NF-κB pathway reversed LPS-induced M1 polarization and balanced the M1/M2 ratio (61, 62). TLR4/MyD88 can phosphorylate MAPKs through the activated TNF receptor associated factor 6 (TRAF6) (39). Therefore, TLR4, as the upstream signal transduction node of MAPKs/NF-κB pathway, has been served as an important target to inhibit MAPKs/NF-κB activation and against neuroinflammation (35).

### 3.5 BDNF/TrkB signaling pathway

BDNF is a neurotrophin exerting a neuroprotective role *via* binding to its high-affinity receptor TrkB (63). BDNF and TrkB are highly expressed in the microglia (64), and play an important role in modulating microglial activation. The upregulated BDNF/TrkB pathway promotes M2 microglial polarization and neurogenesis (65). Activating the BDNF/TrkB pathway also triggers various intracellular signaling pathways (66), such as PI3K/Akt and MAPKs, and has anti-inflammatory properties (67). The PI3K/Akt signaling pathway is the main pathway for TrkB-mediated anti-inflammatory effects (68). The BDNF/TrKB/PI3K/Akt pathway is involved in the mechanisms by which the natural compound curcumin (Cur) inhibits microglial activation induced by traumatic brain injury (TBI) (68). The BDNF/TrkB/ERK pathway has been confirmed to inhibit LPS-induced microglial activation through phosphorylation of the downstream cAMP-response element binding protein (CREB) (64), which is an inhibitor of NF-kB (69).

### 3.6 NLRP3 inflammasome signaling pathway

The NLRP3 inflammasome is a cytoplasmic polyprotein complex existing in microglia constituted by NLRP3, inflammatory protease caspase-1, and the adaptor protein, apoptosis-associated speck-like protein containing a caspase recruitment domain (ASC) (70, 71). The activation of the NLRP3 inflammasome activates caspase-1, promoting pyroptosis as well as the release of proinflammatory cytokines IL-1β and IL-18 (72). The NLRP3 inflammasome is considered a key contributor tomicroglia-mediated neuroinflammation (70, 73). Recent studies indicate that targeting the microglial NLRP3 inflammasome signaling pathway alleviates cognitive abnormalities in POCD (74, 75), and increase treatment efficacy in AD, PD, and TBI (70, 76, 77). Therefore, the NLRP3 inflammasome has become a preventative and therapeutic target for neuroinflammatory diseases.

## 4 Effects of anesthetics on microglial activation *via* signaling pathways

Microglia-mediated neuroinflammation is the critical mechanism in POCD (4, 5). Animal experiments of POCD often use microglial activation as an indicator to assess neuroinflammation and neuronal damage, and it is strongly associated with a decline in cognitive performance (78, 79). Targeting microglia has been proposed as a potential strategy to improve the development of POCD (5). Emerging *in vivo* and *in vitro* evidence (**Tables 1**, **2**) indicates that commonly used anesthetics could target microglial activation through signaling pathways to produce anti- and pro-inflammatory effects, thus ameliorating or exacerbating the development of POCD.

**Table 1:** Effects of anesthetics on microglia and related signaling pathways *in vivo*.

Anesthetic	Anesthetic administration	Animal	Inflammatory model	Cellular/Molecular findings	Signaling pathways	Behavioral findings	Study
Propofol	15mg/kg bolus followed by 1mg/kg/min, iv. with cardiac surgery	20-month-old male rats	Cardiac surgery under propofol or isoflurane anesthesia	↓ microglial activation/↑ miR-223-3p, ↓TNF-α, ↓ IL-1β, ↓IL-6	none	Improving spatial learning and memory	(14)
	2mg/kg bolus followed by 1.3mg/kg/min, i.v. with TBI	Adult male rats	TBI under propofol or isoflurane anesthesia	↓ microglial activation, ↓ neural cell loss	none	Improving reference memory, spatial learning and memory	(17)
	5 mg/kg, i.v. with CFA	4-week-old male mice	CFA injection	↓ microglial activation/↓phosphorylated (p)-ERK1/2, ↓ NF-κB p65	MAPK ERK1/2/NF-κB pathway	Reducing pain hypersensitivity	(80)
	20mg/kg repeated for 2, 4, 6 times, i.p.	P7 male rats	Propofol anesthesia	↑microglial activation/↑caspase-1, ↑IL-1b	NLRP3 inflammasome related pathway	Enhancing locomotoractivity	(13)
	50,75,100,150 mg/kg, i.p.	P7 rats	Propofol anesthesia	↑microglial activation/↓ TrkB, ↓ PI3K, ↓ Akt, ↓ CREB	BDNF/TrkB/PI3K/Akt pathway	No deficits in Morris water maze test	(19)
	15 mg/kg for 4 h, i.g.	Adult male rats	Propofol anesthesia	↓ BDNF, ↑TNF-α, ↑IL-1β, ↑IL-6	BDNF related pathway	Impairing spatial learning and memory	(81)
	200mg/kg daily for 6 days, i.p.	18–20 months old male rats	Propofol anesthesia	↑p-NF-κB p65, ↑NLRP3, ↑caspase-1, ↑TNF-α, ↑IL-1β,↑IL-6	NF-κB pathway and NLRP3 inflammasome pathway	Impairing spatial learning and memory	(82)
Esketamine	5mg/kg, i.p. after laparotomy	7-week-old male mice	laparotomy under 2,2,2-tribromoethanol anesthesia	↓microglial activation/↓ NF-κB p65, ↓ TNF-α, ↓ IL-6	BDNF/TrkB/NF-κB pathway	Improving depression-like behavior	(83)
Ketamine	10, 90 mg/kg, i.p. after LPS injection	9 and 11 weeks old male mice	LPS injection	↓ microglial activation/↓ IL-1 α, ↓ IL-6	none	Improving anxiety-like behavior	(84)
	10mg/kg, i.p. after laparotomy	2-month-old male/female and 16-month-old male mice	laparotomy under isoflurane anesthesia	↑mBDNF, ↑pTrkB	BDNF/TrkB pathway	Improving depression-like behaviors	(85)
	10 mg/kg, i.p. before LPS injection	8-10 weeks old male mice	LPS injection	↓ M1 polarization, ↑M2 polarization/↓ HMGB1, ↓ RAGE	HMGB1/RAGE pathway	Improving depression-like behaviors	(15)
	20 mg/kg, i.p.	P7 male and female rats	Ketamine anesthesia	↑hippocampal pyroptosis/↑NLRP3, ↑caspase-1, ↑IL-1β, ↑IL-18	NLRP3 inflammasome pathway	Impairing spatial learning and memory	(86)
	10, 20, 40, 80 mg/kg, single or six times, i.p.; 30, 60 mg/kg, daily for 6 months, i.p.	2 and 3 months old male mice	Ketamine anesthesia	↑IL-6, ↑IL-1β	none	Inducing spatial memory deficits	(87)
Sevoflurane	3% for 2h	P7 male and femal mice	Sevoflurane anesthesia	↑microglial activation, ↑M1 polarization/↑IL-6, ↑TNF-α, ↑NF-κB	NF-κB pathway	Impairing spatial learning and memory	(88)
	2% for 5h	20‐month‐old male rats	Sevoflurane anesthesia	↑microglial activation/↑IL-6, ↑TNF-α, ↑IL‐1β, ↑p-NF-κB p65	NF-κB pathway	Impairing spatial learning and memory	(89)
	3% for 6 h	16-month-old male mice	Sevoflurane anesthesia	↑ microglial activation/↑NLRP3	NLRP3 inflammasome pathway	Impairing spatial learning and memory	(90)
	3% for 50min with laparotomy	4-month-old female mice	laparotomy under sevoflurane anesthesia	↑microglial activation/↑NLRP3, ↑caspase-1, ↑IL-1β, ↑IL-18	NLRP3 inflammasome pathway	Inducing memory decline	(91)
	2% for 5h	18-20 months old male rats	Sevoflurane anesthesia	↑M1 polarization, ↓ M2 polarization	none	Impairing spatial working memory	(92)
	3% for 2h daily for 3 days	P6 and P60 male and femal mice	Sevoflurane anesthesia	↑ microglial activation	none	Impairing spatial learning and memory	(93)
	3.6% for 6 h	2–3 and 18–20 months old rats	Sevoflurane anesthesia	↑NF-κB p65, ↑TNF-α, ↑IL-1β, ↑IL-6	NF-κB pathway	Impairing age-related spatial learning and memory	(18)
	2.5% for 1 h daily for 5 days prior to the MCAO	8 and 10 weeks old male mice	MCAO under sevoflurane anesthesia	↑M2 polarization ↑p-GSK‐3β, ↑Nrf2	GSK-3β/Nrf2 pathway	improving behaviors in neurobehavioral test	(54)
	2% for 1 h prior to the LPS injection	Adult male mice and rats	LPS injection	↓ microglial activation/↓ IL-6, ↓IL-1β, ↓TNF-α	none	Improving neurocognitive outcomes	(12, 94)
	2% for 15min prior to the MCAO	Rats	MCAO under chloral hydrate anesthesia	↓TLR4, ↓NF-κB p65	TLR4/NF-κB pathway	none	(95)
	2.5% for 4h	P7 male and female mice	Sevoflurane anesthesia	↑neuronal apoptosis/↔IL-1β, ↔IL-6, ↔ TNF-α	none	none	(96)
Isoflurane	1.5% for 2 h	6–8 and 14 months old male mice	Isoflurane anesthesia	↑ microglial activation/↑IL-1β, ↑IL-18, ↑caspase-1 P20	NLRP3 inflammasome pathway	Inducing age-related cognitive decline	(97)
	0.75% for 6h	P7 rats	Isoflurane anesthesia	↑ microglial activation, ↑M1 polarization/↑TLR4, ↑ MyD88, ↑p-NF-κB	TLR4/NF-κB pathway	none	(98)
	1.3% for 6h	8-week-old male mice	Isoflurane anesthesia	↑ microglial activation, ↑M1 polarization/↑CD68, ↑iNOS	none	Inducing cognitive decline	(99)
	1.5% for 4 h	P7 male and female mice	Isoflurane anesthesia	↑neuronal apoptosis/↑IL-1β, ↑IL-6, ↑ TNF-α	none	none	(96)
	2% for 30min prior to the MCAO	Adult male rats	MCAO under chloral hydrate anesthesia	↓microglial activation/↓TLR4,↓MyD88, ↑IκB-α	TLR4/NF-kB pathway	Inproving neurological deficits	(100)
	1.1% or 2.2% for 30min prior to the MCAO	2-month-old male rats	MCAO under isoflurane anesthesia	↓neuronal apoptosis/↑Bcl-2	none	Inproving neurological deficits	(101)
	2% for 30min prior to the MCAO	Adult male rats	MCAO under isoflurane anesthesia	↓neuronal apoptosis/↑p-MAPK P38	MAPK P38 pathway	Inproving neurological deficits	(102)
	2% for 30min prior to the EMP exposure	6-week-old male rats	EMP exposure	↑anti-inflammatory microglia polarization/↑IκB-α, ↓TNF-α, ↓IL-1β, ↓IL-6	NF-kB pathway	none	(103)
Desflurane	6% or 12% for 30min prior to the MCAO	2-month-old male rats	MCAO under desflurane anesthesia	↔Bcl-2	none	No improvement in neurological outcome	(101)
	9% for 2 h daily for 3 days	P6 and P60 male and femal mice	Desflurane anesthesia	↔TNF-α, ↔IL-6	none	No spatial memory impairment	(93)
Lidocaine	1.5mg/kg and maintained with 2 mg/kg/h for 2h, i.v. during isoflurane anesthesia	18-month-old male rats	Isoflurane anesthesia	↓hippocampal cell apoptosis/↔ IL-1β, ↔TNF-α	none	Improving hippocampus-dependent learning and memory.	(104, 105)
	25mg/kg, i.v. after resiniferatoxin injection	6-8 weeks old male rats	Resiniferatoxin injection	↓microglial activation	none	No effects on depression-like behaviors	(106)
	100, 200, and 400 μg/10 μL daily for 7 days, i.t. with morphine	Adult male mice	Morphine injection	↓microglial activation/↓p-MAPK P38 and ↓NF-κB	TLR4/NF-κB pathway and MAPK P38 pathway	none	(107)
	1%, 50ul for 1min, i.t. after CCI	8-10 weeks old male rats	CCI surgery	↓M1 polarization, ↑M2 polarization	none	Reducing neuropathic pain	(16)

↑, promoting effect; ↓, inhibiting effect; ↔, no effect; i.v., intravenous; i.g., intragastric; i.p., intraperitoneal; i.t., intrathecal; TBI, traumatic brain injury; CFA, complete freund’s adjuvant; LPS, lipopolysaccharide; MCAO, middle cerebral artery occlusion; EMP, electromagnetic pulse; CCI, chronic constriction injury; TNF-α, tumor necrosis factor- alpha; IL, interleukin; ERK, extracellular signal-regulated kinase; NF-κB, nuclear factor kappa B; TrkB, tropomyosin receptor kinase B; PI3K, Phosphatidylinositol 3-kinase; Akt, protein kinase B; CREB, cAMP-response element binding protein; BDNF, brain-derived neurotrophic factor; NLRP3, nod-like receptor protein 3; HMGB1, high mobility group box 1; RAGE, receptor for advanced glycation end products; GSK-3β, Glycogen synthase kinase-3 beta; Nrf2, nuclear factor erythroid 2-related factor 2; TLR4, toll-like receptor 4; iNOS, Inducible nitric oxide synthase; MAPK, mitogen-activated protein kinase.

**Table 2:** Effects of anesthetics on microglia and related signaling pathways *in vitro*.

Anesthetics	Anesthetic administration	Cell type	Inflammatory model	Cellular/Molecular findings	Signaling pathways	study
Propofol	50 μM treated with LPS	BV2 cells and Primary microglia	LPS treatment	↓microglial activation/↓NF-κB, ↓ IL-1β, ↓IL-6, ↓TNF-α	NF-κB pathway	(108)
	10,20, 50, 100 µM treated for 2 days after LPS treatment	BV2 cells and Primary microglia	LPS treatment	↓microglial activation/↓NF-κB pathway components (Ticam1, Myd88, Irf3, Nfkb1), ↑ miR-106b, ↓ p-Akt.	NF-κB pathway miR-106b/PI3k/Akt pathway	(109)
	6.25, 12.5, 25, 50, and 100 μM treated for 30min before OGD/R	BV2 cells	OGD/R	↓ microglial activation/↓TLR4, ↓MyD88, ↓NF-κB p65	TLR4/NF-κB pathway	(110)
	30 μM treated for 24h before LPS treatment	BV2 cells	LPS treatment	↓microglial activation/↓TLR4, ↑p-GSK-3β	TLR4 and GSK-3β related pathway	(111)
ketamine	100, 250uM treated for 24h before LPS treatment	Primary microglia	LPS treatment	↓microglial activation/↓p-ERK1/2, ↓NO, ↓IL-1β	MAPK ERK1/2 pathway	(112)
	100uM treated for 15min before LPS treatment	Primary microglia	LPS treatment	↓TNF-α	none	(113)
	none	BV2 cells	LPS treatment	↓ M1 polarization.	HMGB1/RAGE pathway	(15)
	25, 50, 100, 150uM treated for 6h	Human microglia cells	Ketamine treatment	↑ M1 polarization, ↑neural cell death	none	(114)
Sevoflurane	2.0% for 5h	BV2 cells	Sevoflurane treatment	↑ M1 polarization, ↓M2 polarization	none	(115)
	2.5% for 1h before OGD	Primary microglia	OGD	↑M2 polarization/↑ p-GSK‐3β, ↑Nrf2	GSK-3β/Nrf2 pathway	(54)
Isoflurane	2% for 6h	Primary microglia	Isoflurane treatment	↑nuclear NF-κB	NF-κB pathway	(116)
	3% for 24h	BV2 cells	Isoflurane treatment	↑microglial activation/↑NLRP3, ↑IL-1β, ↑IL-18	NLRP3 inflammasome pathway	(117)
	2% for 6h after LPS treatment	Primary microglia	LPS treatment	↑NLRP3, ↑IL-1β, ↑IL-18	NLRP3 inflammasome pathway	(97)
	0.4% for 6h	BV2 cells	Isoflurane treatment	↑microglial activation/↑IL-1β, ↑TNF-α, ↑IL-6, ↑TLR-4	TLR4 related pathway	(98)
Lidocaine	0.1 mM, 1 mM, or 10 mM treated for 2h with ATP	Primary microglia	ATP treatment	↓p-MAPK p38, ↓TNF-α, ↓IL-1β, ↓IL-6	MAPK P38 pathway	(118)
	0.2, 2, and 20 μg/mL treated for 1h before LPS treatment	Primary microglia	LPS treatment	↓p-MAPK p38, ↓PGE2, ↓TNF-α, ↓ IL-1β	MAPK P38 pathway	(119)
	10ug/ml treated for 24h with LPS	HAPI microglia cell line	LPS or IL-4 treatment	↓ M1 polarization, ↑ M2 polarization	none	(16)

↑, promoting effect; ↓, inhibiting effect; ↔, no effect; OGD/R, oxygen-glucose deprivation/reoxygenation; ATP, adenosine triphosphate; HAPI, highly aggressively proliferating immortalized; Ticam1, toll-like receptor adaptor molecule 1; Myd88, myeloid differentiation primary response 88; Irf3, interferon regulatory factor 3; Nfkb1, nuclear factor kappa B subunit 1; NO, nitric oxide; PGE2, prostaglandin E2.

### 4.1 Intravenous anesthetics

#### 4.1.1 Propofol

Propofol is a short-acting intravenous anesthetic commonly used for anesthesia induction and maintenance (120). It potentiates the gamma-aminobutyric acid A (GABAA) receptors while blocking the N-methyl-D-aspartate (NMDA) receptors (120). Clinical evidence suggests that propofol could be beneficial in reducing elderly POCD incidence (121, 122), as the anti-inflammatory property of propofol. Aged rats with cardiac surgery under propofol anesthesia showed less neuroinflammation and improved cognitive outcomes because of attenuated microglial activation (14). Similarly, in the TBI model with significant neuroinflammation, the administration of propofol inhibits microglial activation and attenuates neuronal cell death, thus improving cognitive recovery after brain injury (17). Therefore, propofol is a potential anesthetic with neuroprotective properties through suppressing microglial activation.

Accumulating evidence has confirmed that propofol targets NF-κB and its upstream signaling pathways to inhibit microglial activation *in vivo* and *in vitro* (80, 108–110). Microglial activation in the spinal cord induced by peripheral inflammation can be reversed by propofol *via* inhibition of the MAPK ERK1/2/NF-κB pathway (80). In the LPS-induced cell model, the release of pro-inflammatory cytokines and the genes TICAM1, IRF3, and NFKB1 involved in NF-κB pathway are downregulated by propofol (108, 109). TLR4 and its adaptor protein MyD88, key upstream inflammatory mediators that activate NF-κB, are also downregulated by propofol, thus inhibiting the microglial activation induced by LPS (110, 111).

The PI3k/Akt pathway is also involved in the anti-inflammatory mechanisms of propofol on microglial activation (109, 123). Liu et al. (109) found that miRNA miR-106b acted as an upstream anti-inflammatory regulator, inhibiting Akt phosphorylation; propofol induced the overexpression of miR-106b to attenuate LPS-induced microglial activation by inhibiting the PI3k/Akt pathway (109). In addition, GSK-3β, the activation of which facilitates neuroinflammation, can be inactivated by Akt (123) and is also inactivated by propofol in LPS-treated BV2 cells, and this may be related to the activation of PI3k/Akt pathway (111). Together, multiple signaling pathways, including the NF-κB pathway, with the upstream MAPK and TLR4/MyD88 signaling, and the PI3k/Akt pathway, are involved in the anti-inflammatory mechanisms of propofol on microglia. This provides important evidence for the potential benefits of propofol on POCD. Could propofol be recommended as a preventative therapy for POCD?

Unfortunately, propofol is not entirely anti-inflammatory and neuroprotective. Propofol also promotes neuroinflammation and microglial activation in an age-dependent manner (13, 19). Propofol-induced neuroinflammation occurs primarily in the developing brain, vulnerable due to extensive synaptogenesis, resulting in developmental neurotoxicity (124, 125). Propofol administration induces microglial activation and neuroinflammation in P7 neonatal rats (13, 19). However, the mechanism by which propofol promotes microglial activation in the developing brain is not completely clear. Studies indicate it is related to the downregulated hippocampal neurotrophin BDNF, thus inhibiting BDNF/TrKB signaling and downstream PI3K/Akt activation (19, 81). Importantly, the pro-inflammatory effect of propofol is closely related to exposure time. Repeated administration of propofol in both neonatal and aged rats led to long-term cognitive injury as well as the upregulation of NF-κB and NLRP3 inflammasome in the brain (13, 82), suggesting the activation of NF-κB and NLRP3 inflammasome pathways participate in the pro-inflammatory mechanisms of propofol. Further validation of the effects of repeated propofol exposure on microglial activation, which is the key to neuroinflammation development, is required.

#### 4.1.2 Ketamine

Ketamine and its more potent S-enantiomer (esketamine) work as NMDA-receptor antagonists, benefiting from their short-term anesthesia and analgesia, which are frequently used for pediatric anesthesia and for procedure sedations outside the operating room (126, 127). The effects of ketamine on POCD remains controversial. Clinical research suggesting an alleviated impact of ketamine on cognitive impairment following cardiac surgery is inconclusive (128–130), and this may be the result of ketamine’s anti- and pro-inflammatory effects.

Several studies have elucidated the anti-inflammatory properties of ketamine, notably in neuroinflammation-induced depression (84, 131). Ketamine’s anti-inflammatory role is primarily associated with the suppression of microglial activation (84, 112). Shibakawa et al. (113) found ketamine, more so than propofol, inhibited the release of TNFα in LPS-treated microglia cells. It was confirmed *in vitro* that ketamine inhibits microglial activation through the MAPK ERK1/2 pathway (112). In addition, blockade of the glutamate NMDA receptors by ketamine or esketamine induces binding between glutamate and α-amino-3-hydroxy-5-methyl-4-isoxazolepropionic acid (AMPA) receptors, leading to synaptic release of BDNF, which activates the TrkB pathway and participates in NF-κB translocation inhibition (85). Esketamine decreases the number of activated microglia cells and improves depression-like behaviors in a postoperative depression (POD) model, *via* the BDNF/TrkB/NF-κB signaling pathway (83). Moreover, the administration of ketamine reverses microglial M1 polarization induced by LPS and promotes M2 polarization *in vivo* and *in vitro* in association with the downregulated HMGB1/RAGE axis (15). The HMGB1/RAGE axis activates NF-κB, and its inhibition can induce neuroprotective effects (40, 132).

Recent evidence suggests ketamine’s potential targeting of microglial activation may explain its pro-inflammatory role (86, 87, 114), but studies remain insufficient. A recent *in vitro* study suggests that ketamine administration induces microglial M1 polarization, thus increasing neural cell death (114). However, the mechanism by which ketamine increases the M1 phenotype remains unknown. In terms of ketamine’s pro-inflammatory effect, it has been suggested that ketamine-induced neuroinflammation depends on dose and exposure time. A high dose of ketamine (80 mg/kg) or long-term exposure for 6 months can aggravate neuroinflammation and impair neurocognitive performance (87). In addition, like propofol, developmental neurotoxicity is also induced by ketamine (86, 133). The cognitive deficits in P7 neonatal rats induced by clinical doses of ketamine (20 mg/kg) are associated with hippocampal NLRP3 inflammasome activation (86). In short, the anti-inflammatory properties of ketamine might transform into pro-inflammatory properties, depending on dose, exposure time, and age. It is thus crucial to identify the dose-, exposure time-, and age-dependent effects of ketamine on microglial activation and elucidate the pro-inflammatory mechanisms of ketamine.

### 4.2 Volatile anesthetics

#### 4.2.1 Sevoflurane

Sevoflurane, the most widely used volatile anesthetic agent, can exhibit both anti- and pro-inflammatory properties (12, 54, 134). Sevoflurane-induced neuroinflammation plays a major role in the pathogenesis of POCD and has been well-explored (11, 134). Several animal studies suggest the neurocognitive dysfunction induced by sevoflurane is related to microglial activation and microglial M1 polarization *via* the NF-kB pathway (88–90). *In vivo* and *in vitro* studies have identified sevoflurane as suppressing microglial M2 polarization in the process of neuroinflammation (92, 115), thereby aggravating neural injury development. This suggests the imbalance of the M1/M2 microglia ratio is the central mechanism involved in sevoflurane-induced neuroinflammation. A recent study by Tang et al. (88) showed that resveratrol, a polyphenolic compound, reverses the imbalance of the M1/M2 microglia ratio in sevoflurane-exposed neonatal mice *via* the NF-kB pathway. Similarly, carnosol, a natural ingredient, can inhibit sevoflurane-induced microglial activation through the NF-kB pathway in aged rats (89). In addition, the NLRP3 inflammasome pathway is upregulated by sevoflurane with or without surgery, and induces abnormal microglial activation (90, 91). Inhibiting the NLRP3 inflammasome in activated microglia has produced beneficial reduction in cognitive deficits (90, 91). Thus, the microglial NF-kB and NLRP3 inflammasome pathways could be potential targets for the intervention of sevoflurane-induced neuroinflammation.

The pro-inflammatory effects of sevoflurane are age-dependent, manifesting primarily in neonatal and elderly individuals (18, 93). Previous studies show that the neuroinflammation and cognitive impairment induced by sevoflurane occurs in neonatal and old-age mice, but not adult mice (18, 93). Moreover, several clinical trials suggest that sevoflurane exposure caused neurocognitive deficits in elderly surgical patients (121, 135). The pro-inflammatory effect of sevoflurane also depends on exposure time. Microglial activation is directly associated with long-term exposure to sevoflurane, e.g., 5-6 h of exposure (89, 90, 92). Therefore, clinical procedures that require prolonged anesthesia, especially in newborns and elderly3patients, require extra attention to the potential neuroinflammation and postoperative cognitive impairment induced by sevoflurane.

Although the neurotoxicity of sevoflurane has been described in several studies, the neuroprotection of sevoflurane is still discovered, and known to be dose-dependent (12, 54, 94, 136). The anti-inflammatory mechanisms of sevoflurane have been well explored in cerebral I/R injury models (137). A sub-anesthetic dose of sevoflurane (2.5%) preconditioning engages M2 microglia polarization *via* the GSK-3β phosphorylation and Nrf2 activation (54), which contributes to the M1/M2 microglial phenotype shift (55). A lower dose of sevoflurane (2.0%) administration inhibited LPS-induced microglial activation (12, 94) and the release of proinflammatory cytokines after cerebral I/R injury *via* the TLR4/NF-κB pathway (95). In addition, low-dose sevoflurane (1.3% and 1.8%) promoted hippocampal neurogenesis and enhance spatial learning memory in neonatal rats (136, 138). Based on these studies, the low dose of sevoflurane may be responsible for its anti-inflammatory effects. Assessing the neuroprotective threshold concentration of sevoflurane is there necessary.

#### 4.2.2 Isoflurane

Another volatile anesthetic, isoflurane, has pro-inflammatory effects similar to sevoflurane. Both the microglial NF-κB and NLRP3 pathways are major agonist pathways for isoflurane-induced neuroinflammation (97, 98, 116, 117). Additionally, prolonged isoflurane inhalation for 6 h leads to microglial activation and M1 polarization *via* the upregulation of TLR4/NF-κB pathway (98, 99), and cognitive impairment correlates directly with the multiple isoflurane exposures (139). One study comparing the pro-inflammatory properties of isoflurane with sevoflurane, discovered that at equivalent doses, isoflurane induces a significantly greater neuroinflammatory response (96), although whether this leads to more severe cognitive impairment remains to be explored.

Isoflurane also has neuroprotective benefits, suppressing microglial overactivation and reducing neuroinflammatory response. Short-term isoflurane pretreatment for 30 min reduces infarct size and enhances neurological function in the cerebral I/R model (100–102), and isoflurane reduces both microglial activation and neuronal apoptosis in infarct foci *via* the TLR4/NF-κB pathway (100, 101). In a model of electromagnetic pulse (EMP) exposure that triggers neuroinflammation and microglial activation, 30 min isoflurane pretreatment shifts microglia from pro-inflammatory to anti-inflammatory phenotype by significantly upregulating IκB-α, an inhibitor of NF-κB (103). It is thus possible that a temporary application of isoflurane may have neuroprotective benefits. However, to date, the neuroprotective mechanism of isoflurane and sevoflurane pretreatment have been primarily seen in the cerebral I/R model, which cannot rule out disease model specificity, and additional validation in the POCD model is required.

The volatile anesthetic desflurane, which has the benefit of rapid elimination, has been studied comparatively little in the pathogenesis of POCD. Clinical evidence suggests that desflurane may be superior to sevoflurane and isoflurane in reducing cognitive decline after surgery (140, 141). Previous studies found that multiple exposures to desflurane do not lead to cognitive impairment and neuroinflammation (93). However, neuroprotective effects were not observed following pretreatment with desflurane prior to cerebral I/R injury (101). Further studies are necessary to discover which volatile anesthetic improves patient safety the most.

### 4.3 Local anesthetics

Lidocaine, as the most used amino-amide local anesthetic, is well-known for its anti-inflammatory and neuroprotective properties (142–144). Clinical trials indicate that a continuous intravenous infusion of lidocaine for 48 h can reduce the cerebral inflammatory response induced by cardiopulmonary bypass (CPB) (144). A meta-analysis suggests that the perioperative administration of lidocaine was protective against POCD occurrence following cardiac surgery (143). The neuroprotective properties of lidocaine have also been observed in POCD rats (104, 105). *In vivo* and *in vitro* studies indicate that anti-inflammatory mechanisms of lidocaine include the inhibition of microglial activation *via* the TLR4/NF-κB and MAPK P38 pathways (106, 107, 118, 119). In addition, a recent study showed that the administration of lidocaine can alleviate microglial activation by inhibiting M1 polarization while increasing abundance of the anti-inflammatory M2 phenotype in the microglial line HAPI cells, and this was also confirmed in the rat neuropathic pain model (16). However, the regulatory mechanism of lidocaine on the M1/M2 ratio awaits further exploration.

Although the anti-inflammatory and neuroprotective effects of lidocaine are well supported, some clinical studies have failed to confirm that lidocaine reduces the occurrence of POCD (145, 146). Individual animal studies suggest that lidocaine injections induce neuroinflammation, possibly in association with the promotion of other immune cell activation (147). Therefore, the inhibitory effect of lidocaine on neuroinflammation remains inconclusive and warrants further investigation. Furthermore, since the inhibitory effect on microglial activation has been identified mostly in a neuropathic pain model, further validation in the POCD model is necessary.

## 5 Discussion

**Figure 1:**
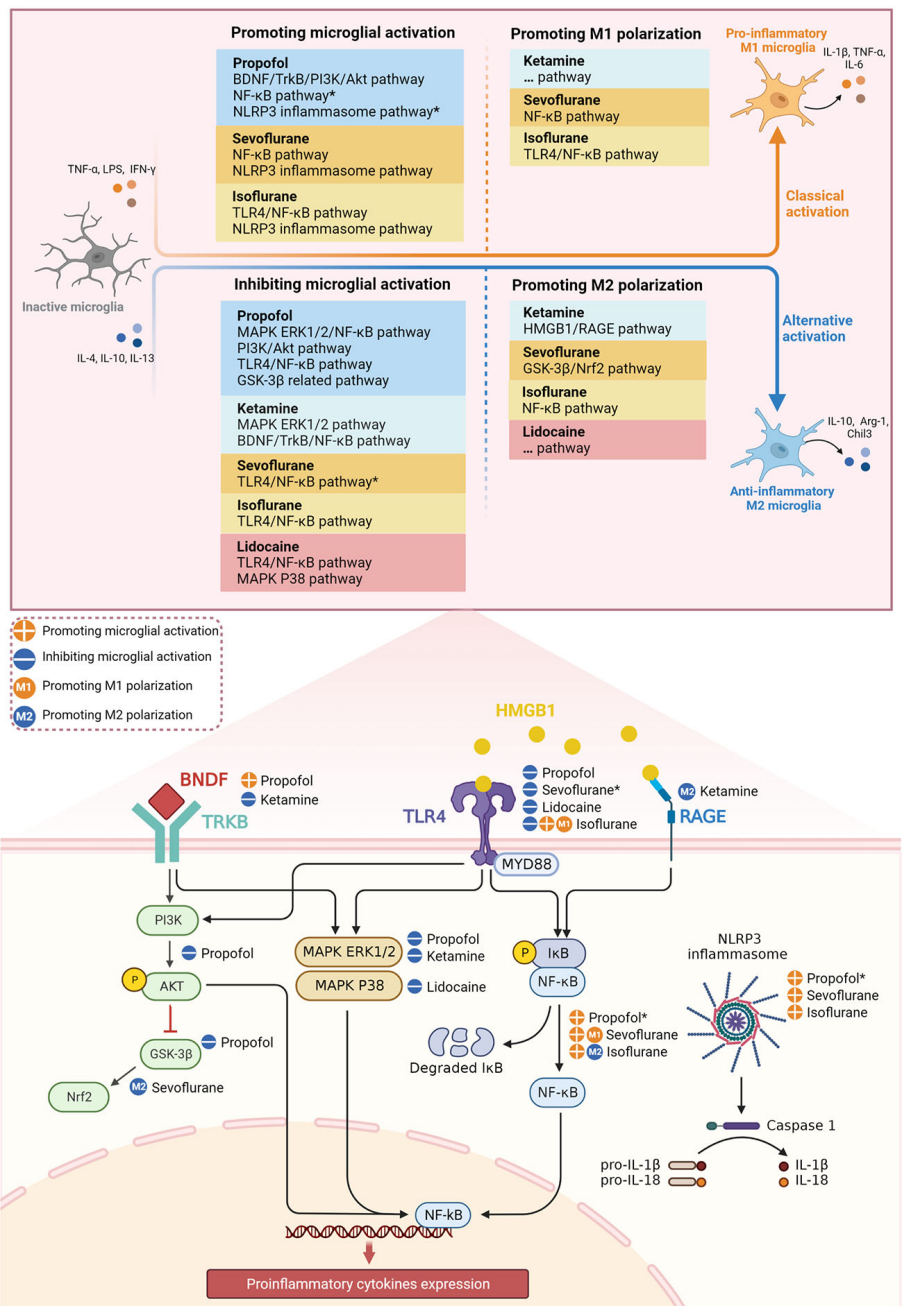
Effect of different anesthetics on microglial activation and M1/M2 polarization through inflammatory signaling pathways. *The potential signaling pathways involved in the mechanism of anesthetics on microglial activation.

In recent years, the role of anesthetics in POCD has been extensively studied, yet it continues to be controversial. In this review, we summarize the effects of anesthetics on microglial activation and M1/M2 polarization *via* multiple inflammatory signaling pathways (**Figure 1**). We focus on the possible dual beneficial and detrimental effects of anesthetics in POCD by targeting microglia with anti- and pro-inflammatory properties. In terms of evaluating the potential of anesthetics to ameliorate POCD based on their anti-inflammatory properties, we conclude the following.

First, the intravenous anesthetics propofol and ketamine show significant anti-inflammatory and neuroprotective effects (14, 17, 84), but the neuroinflammation and cognitive impairment induced by long-term administration (82, 87), and especially developmental neurotoxicity (133), cannot be ignored. Mechanistically, the pro-inflammatory effects of propofol may be associated with downregulated BDNF/TrkB/PI3K/Akt pathway (19, 81), as well as activated NF-kB and NLRP3 inflammasome pathways in microglia (13, 82). Recent studies demonstrate that ketamine promotes microglial M1 polarization (114), but the exact mechanism by which this occurs is unclear and needs further exploration. Furthermore, ketamine as a novel antidepressant (148), the anti-inflammatory mechanism of which has mostly been studied using depression models (15, 84), also requires further validation in POCD models.

Second, the volatile anesthetics sevoflurane and isoflurane have similar pro-inflammatory mechanisms, but their distinct pro-inflammatory properties may result in different degrees of cognitive impairment. It is of interest that, in cerebral I/R injury models, pretreatment with low-dose sevoflurane or short-term isoflurane can both suppress microglial activation, which may be *via* the TLR4/NF-κB pathway (95, 100). Sevoflurane and isoflurane also promote microglial M2 polarization by activating Nrf2 and inhibiting NF-κB, respectively (54, 103). In addition, the rapid elimination of desflurane may explain why it is less likely to cause neuroinflammation and cognitive impairment, although the exact mechanism remains to be studied. These studies reveal the neuroprotective potential of volatile anesthetics, an important direction for future research to reduce POCD.

Third, lidocaine, a commonly used local anesthetic, has been suggested to reduce the occurrence of POCD after cardiac surgery (143), but contradictory results remain (145, 146). Current evidence suggests lidocaine has significant anti-inflammatory effects and facilitates microglial M2 polarization (16, 106, 107, 118); however, the related signaling pathways need further exploration. It has also been suggested that lidocaine may induce neuroinflammation and be associated with the activation of other CNS immune cells (147), and that its pro-inflammatory effects are complex and require further study. Moreover, it is important to note that these mechanistic studies of anesthetics need to be combined with clinical studies in the future to obtain more convincing conclusions.

In conclusion, anesthetics are a double-edged sword for POCD. High doses, prolonged exposure time, and the vulnerable phase of newborns and elderly patients may lead to a shift from a beneficial impact of anesthetics on POCD toward worsening outcomes. The selection of appropriate anesthetic drugs will always be a challenge for anesthesiologists, but the anti-inflammatory properties of anesthetic drugs provide promise in helping to reduce the incidence of POCD and more in-depth studies are urgently needed.

## Author contributions

YY conceived and designed the study. MZ selected the articles and wrote the first draft of the manuscript. YY and MZ contributed to manuscript revision, read, and approved the submitted version. All authors contributed to the article and approved the submitted version.

## References

[1] Moller JT Cluitmans P Rasmussen LS Houx P Rasmussen H Canet J. Long-term postoperative cognitive dysfunction in the elderly: ISPOCD1 study. Lancet (1998) 351:857–61. doi: 10.1016/S0140-6736(97)07382-0 9525362

[2] Jungwirth B Zieglgaensberger W Kochs E Rammes G. Anesthesia and postoperative cognitive dysfunction (POCD). Mini-Rev Med Chem (2009) 9:1568–79. doi: 10.2174/138955709791012229 20088778

[3] Monk TG Weldon BC Garvan CW Dede DE van der Aa MT Heilman KM. Predictors of cognitive dysfunction after major noncardiac surgery. Anesthesiology (2008) 108:18–30. doi: 10.1097/01.anes.0000296071.19434.1e 18156878

[4] Safavynia SA Goldstein PA. The role of neuroinflammation in postoperative cognitive dysfunction: Moving from hypothesis to treatment. Front Psychiatry (2019) 9:752. doi: 10.3389/fpsyt.2018.00752 30705643 PMC6345198

[5] Feng X Valdearcos M Uchida Y Lutrin D Maze M Koliwad SK. Microglia mediate postoperative hippocampal inflammation and cognitive decline in mice. JCI Insight (2017) 2:e91229. doi: 10.1172/jci.insight.91229 28405620 PMC5374063

[6] Guo S Wang H Yin Y. Microglia polarization from M1 to M2 in neurodegenerative diseases. Front Aging Neurosci (2022) 14:815347. doi: 10.3389/fnagi.2022.815347 35250543 PMC8888930

[7] Kettenmann H Hanisch U-K Noda M Verkhratsky A. Physiology of microglia. Physiol Rev (2011) 91:461–553. doi: 10.1152/physrev.00011.2010 21527731

[8] Orihuela R McPherson CA Harry GJ. Microglial M1/M2 polarization and metabolic states. Br J Pharmacol (2016) 173:649–65. doi: 10.1111/bph.13139 PMC4742299 25800044

[9] Tang Y Le W. Differential roles of M1 and M2 microglia in neurodegenerative diseases. Mol Neurobiol (2016) 53:1181–94. doi: 10.1007/s12035-014-9070-5 25598354

[10] Evered LA Silbert BS. Postoperative cognitive dysfunction and noncardiac surgery. Anesth Analg (2018) 127:496–505. doi: 10.1213/ANE.0000000000003514 29889707

[11] Wang C Chen W Zhang Y Lin S He H. Update on the mechanism and treatment of sevoflurane-induced postoperative cognitive dysfunction. Front Aging Neurosci (2021) 13:702231. doi: 10.3389/fnagi.2021.702231 34305576 PMC8296910

[12] Satomoto M Sun Z Adachi YU Kinoshita H Makita K. Sevoflurane preconditioning ameliorates lipopolysaccharide-induced cognitive impairment in mice. Exp Anim (2018) 67:193–200. doi: 10.1538/expanim.17-0102 29187700 PMC5955751

[13] Milanovic D Pesic V Loncarevic-Vasiljkovic N Pavkovic Z Popic J Kanazir S. The fas Ligand/Fas death receptor pathways contribute to propofol-induced apoptosis and neuroinflammation in the brain of neonatal rats. Neurotox Res (2016) 30:434–52. doi: 10.1007/s12640-016-9629-1 27189477

[14] Lian F Cao C Deng F Liu C Zhou Z. Propofol alleviates postoperative cognitive dysfunction by inhibiting inflammation *via* up-regulating miR-223-3p in aged rats. Cytokine (2022) 150:155783. doi: 10.1016/j.cyto.2021.155783 34979347

[15] Wu M Zhao L Wang Y Guo Q An Q Geng J. Ketamine regulates the autophagy flux and polarization of microglia through the HMGB1-RAGE axis and exerts antidepressant effects in mice. J Neuropathol Exp Neurol (2022) 81:931–42. doi: 10.1093/jnen/nlac035 35582883

[16] Yuan J Fei Y. Lidocaine ameliorates chronic constriction injury-induced neuropathic pain through regulating M1/M2 microglia polarization. Open Med (2022) 17:897–906. doi: 10.1515/med-2022-0480 PMC9106111 35647302

[17] Luo T Wu J Kabadi SV Sabirzhanov B Guanciale K Hanscom M. Propofol limits microglial activation after experimental brain trauma through inhibition of nicotinamide adenine dinucleotide phosphate oxidase. Anesthesiology (2013) 119:1370–88. doi: 10.1097/ALN.0000000000000020 24121215

[18] Yang Z Yuan C. IL-17A promotes the neuroinflammation and cognitive function in sevoflurane anesthetized aged rats *via* activation of NF-b signaling pathway. BMC Anesthesiol (2018) 18:147. doi: 10.1186/s12871-018-0607-4 30342469 PMC6195755

[19] Yang Y Yi J Pan M Hu B Duan H. Edaravone alleviated propofol-induced neural injury in developing rats by BDNF/TrkB pathway. J Cell Mol Med (2021) 25:4974–87. doi: 10.1111/jcmm.16422 PMC8178254 33932098

[20] Dresselhaus EC Meffert MK. Cellular specificity of NF-kappa b function in the nervous system. Front Immunol (2019) 10:1043. doi: 10.3389/fimmu.2019.01043 31143184 PMC6520659

[21] Wright-Jin EC Gutmann DH. Microglia as dynamic cellular mediators of brain function. Trends Mol Med (2019) 25:967–79. doi: 10.1016/j.molmed.2019.08.013 PMC6829057 31597593

[22] Parkhurst CN Yang G Ninan I Savas JN Yates JR Lafaille JJ. Microglia promote learning-dependent synapse formation through brain-derived neurotrophic factor. Cell (2013) 155:1596–609. doi: 10.1016/j.cell.2013.11.030 PMC4033691 24360280

[23] Haruwaka K Ikegami A Tachibana Y Ohno N Konishi H Hashimoto A. Dual microglia effects on blood brain barrier permeability induced by systemic inflammation. Nat Commun (2019) 10:5816. doi: 10.1038/s41467-019-13812-z 31862977 PMC6925219

[24] Woodburn SC Bollinger JL Wohleb ES. The semantics of microglia activation: neuroinflammation, homeostasis, and stress. J Neuroinflamm (2021) 18:258. doi: 10.1186/s12974-021-02309-6 PMC8571840 34742308

[25] Colonna M Butovsky O. Microglia function in the central nervous system during health and neurodegeneration. In: Littman DR Yokoyama WM, editors. Annual review of immunology, vol. 35 . Palo Alto: Annual Reviews (2017). p. 441–68. doi: 10.1146/annurev-immunol-051116-052358 PMC8167938 28226226

[26] Wang J Xing H Wan L Jiang X Wang C Wu Y. Treatment targets for M2 microglia polarization in ischemic stroke. BioMed Pharmacother (2018) 105:518–25. doi: 10.1016/j.biopha.2018.05.143 29883947

[27] Brown GC Vilalta A. How microglia kill neurons. Brain Res (2015) 1628:288–97. doi: 10.1016/j.brainres.2015.08.031 26341532

[28] Kanazawa M Ninomiya I Hatakeyama M Takahashi T Shimohata T. Microglia and Monocytes/Macrophages polarization reveal novel therapeutic mechanism against stroke. Int J Mol Sci (2017) 18:2135. doi: 10.3390/ijms18102135 29027964 PMC5666817

[29] Wan T Huang Y Gao X Wu W Guo W. Microglia polarization: A novel target of exosome for stroke treatment. Front Cell Dev Biol (2022) 10:842320. doi: 10.3389/fcell.2022.842320 35356292 PMC8959940

[30] Tay TL Savage JC Hui CW Bisht K Tremblay M-E. Microglia across the lifespan: from origin to function in brain development, plasticity and cognition. J Physiol-London (2017) 595:1929–45. doi: 10.1113/JP272134 PMC5350449 27104646

[31] Popiolek-Barczyk K Mika J. Targeting the microglial signaling pathways: New insights in the modulation of neuropathic pain. Curr Med Chem (2016) 23:2908–28. doi: 10.2174/0929867323666160607120124 PMC5427777 27281131

[32] Leng F Edison P. Neuroinflammation and microglial activation in Alzheimer disease: where do we go from here? Nat Rev Neurol (2021) 17:157–72. doi: 10.1038/s41582-020-00435-y 33318676

[33] Jin X Liu M-Y Zhang D-F Zhong X Du K Qian P. Baicalin mitigates cognitive impairment and protects neurons from microglia-mediated neuroinflammation *via* suppressing NLRP3 inflammasomes and TLR4/NF-kappa b signaling pathway. CNS Neurosci Ther (2019) 25:575–90. doi: 10.1111/cns.13086 PMC6488900 30676698

[34] Kumar V. Toll-like receptors in the pathogenesis of neuroinflammation. J Neuroimmunol (2019) 332:16–30. doi: 10.1016/j.jneuroim.2019.03.012 30928868

[35] Rahimifard M Maqbool F Moeini-Nodeh S Niaz K Abdollahi M Braidy N. Targeting the TLR4 signaling pathway by polyphenols: A novel therapeutic strategy for neuroinflammation. Ageing Res Rev (2017) 36:11–9. doi: 10.1016/j.arr.2017.02.004 28235660

[36] Kawai T Adachi O Ogawa T Takeda K Akira S. Unresponsiveness of MyD88-deficient mice to endotoxin. Immunity (1999) 11:115–22. doi: 10.1016/S1074-7613(00)80086-2 10435584

[37] Rosadini CV Kagan JC. Early innate immune responses to bacterial LPS. Curr Opin Immunol (2017) 44:14–9. doi: 10.1016/j.coi.2016.10.005 PMC5426986 27842237

[38] Xu S Wang J Jiang J Song J Zhu W Zhang F. TLR4 promotes microglial pyroptosis *via* lncRNA-F630028O10Rik by activating PI3K/AKT pathway after spinal cord injury. Cell Death Dis (2020) 11:693. doi: 10.1038/s41419-020-02824-z 32826878 PMC7443136

[39] Takeda K Akira S. TLR signaling pathways. Semin Immunol (2004) 16:3–9. doi: 10.1016/j.smim.2003.10.003 14751757

[40] Fan H Tang H-B Chen Z Wang H-Q Zhang L Jiang Y. Inhibiting HMGB1-RAGE axis prevents pro-inflammatory macrophages/microglia polarization and affords neuroprotection after spinal cord injury. J Neuroinflamm (2020) 17:295. doi: 10.1186/s12974-020-01973-4 PMC7547440 33036632

[41] Sims GP Rowe DC Rietdijk ST Herbst R Coyle AJ. HMGB1 and RAGE in inflammation and cancer. In: Paul WE Littman DR Yokoyama WM, editors. Annual review of immunology, vol. 28 . Palo Alto: Annual Reviews (2010). p. 367–88. doi: 10.1146/annurev.immunol.021908.132603 20192808

[42] Yeh FL Hansen DV Sheng M. TREM2, microglia, and neurodegenerative diseases. Trends Mol Med (2017) 23:512–33. doi: 10.1016/j.molmed.2017.03.008 28442216

[43] Chen S Peng J Sherchan P Ma Y Xiang S Yan F. TREM2 activation attenuates neuroinflammation and neuronal apoptosis *via* PI3K/Akt pathway after intracerebral hemorrhage in mice. J Neuroinflamm (2020) 17:168. doi: 10.1186/s12974-020-01853-x PMC7257134 32466767

[44] Han X Cheng X Xu J Liu Y Zhou J Jiang L. Activation of TREM2 attenuates neuroinflammation *via* PI3K/Akt signaling pathway to improve postoperative cognitive dysfunction in mice. Neuropharmacology (2022) 219:109231. doi: 10.1016/j.neuropharm.2022.109231 36041498

[45] Lawrence T. The nuclear factor NF-kappa b pathway in inflammation. Cold Spring Harbor Perspect Biol (2009) 1:a001651. doi: 10.1101/cshperspect.a001651 PMC2882124 20457564

[46] Meffert MK Chang JM Wiltgen BJ Fanselow MS Baltimore D. NF-kappa b functions in synaptic signaling and behavior. Nat Neurosci (2003) 6:1072–8. doi: 10.1038/nn1110 12947408

[47] Shih R-H Wang C-Y Yang C-M. NF-kappaB signaling pathways in neurological inflammation: A mini review. Front Molec Neurosci (2015) 8:77. doi: 10.3389/fnmol.2015.00077 26733801 PMC4683208

[48] Paudel YN Shaikh MF Chakraborti A Kumari Y Aledo-Serrano A Aleksovska K. HMGB1: A common biomarker and potential target for TBI, neuroinflammation, epilepsy, and cognitive dysfunction. Front Neurosci (2018) 12:628. doi: 10.3389/fnins.2018.00628 30271319 PMC6142787

[49] Alam A Hana Z Jin Z Suen KC Ma D. Surgery, neuroinflammation and cognitive impairment. EBioMedicine (2018) 37:547–56. doi: 10.1016/j.ebiom.2018.10.021 PMC6284418 30348620

[50] Gan P Ding L Hang G Xia Q Huang Z Ye X. Oxymatrine attenuates dopaminergic neuronal damage and microglia-mediated neuroinflammation through cathepsin d-dependent HMGB1/TLR4/NF-kappa b pathway in parkinson’s disease. Front Pharmacol (2020) 11:776. doi: 10.3389/fphar.2020.00776 32528295 PMC7264119

[51] Cianciulli A Porro C Calvello R Trotta T Lofrumento DD Panaro MA. Microglia mediated neuroinflammation: Focus on PI3K modulation. Biomolecules (2020) 10:137. doi: 10.3390/biom10010137 31947676 PMC7022557

[52] Chu E Mychasiuk R Hibbs ML Semple BD. Dysregulated phosphoinositide 3-kinase signaling in microglia: shaping chronic neuroinflammation. J Neuroinflamm (2021) 18:276. doi: 10.1186/s12974-021-02325-6 PMC8627624 34838047

[53] Zheng K Lv B Wu L Wang C Xu H Li X. Protecting effect of emodin in experimental autoimmune encephalomyelitis mice by inhibiting microglia activation and inflammation *via* Myd88/PI3K/Akt/NF-kappa b signalling pathway. Bioengineered (2022) 13:9322–44. doi: 10.1080/21655979.2022.2052671 PMC9161934 35287559

[54] Cai M Sun S Wang J Dong B Yang Q Tian L. Sevoflurane preconditioning protects experimental ischemic stroke by enhancing anti-inflammatory microglia/macrophages phenotype polarization through GSK-3 beta/Nrf2 pathway. CNS Neurosci Ther (2021) 27:1348–65. doi: 10.1111/cns.13715 PMC8504524 34370899

[55] Lei X Li H Li M Dong Q Zhao H Zhang Z. The novel Nrf2 activator CDDO-EA attenuates cerebral ischemic injury by promoting microglia/macrophage polarization toward M2 phenotype in mice. CNS Neurosci Ther (2021) 27:82–91. doi: 10.1111/cns.13496 33280237 PMC7804925

[56] Cargnello M Roux PP. Activation and function of the MAPKs and their substrates, the MAPK-activated protein kinases. Microbiol Mol Biol Rev (2011) 75:50–83. doi: 10.1128/MMBR.00031-10 21372320 PMC3063353

[57] Li Z Chi H Zhu W Yang G Song J Mo L. Cadmium induces renal inflammation by activating the NLRP3 inflammasome through ROS/MAPK/NF-kappa b pathway *in vitro* and *in vivo* . Arch Toxicol (2021) 95:3497–513. doi: 10.1007/s00204-021-03157-2 34510229

[58] Liu Z Yao X Jiang W Li W Zhu S Liao C. Advanced oxidation protein products induce microglia-mediated neuroinflammation *via* MAPKs-NF-kappa b signaling pathway and pyroptosis after secondary spinal cord injury. J Neuroinflamm (2020) 17:90. doi: 10.1186/s12974-020-01751-2 PMC7082940 32192500

[59] Qin S Yang C Huang W Du S Mai H Xiao J. Sulforaphane attenuates microglia-mediated neuronal necroptosis through down-regulation of MAPK/NF-kappa b signaling pathways in LPS-activated BV-2 microglia. Pharmacol Res (2018) 133:218–35. doi: 10.1016/j.phrs.2018.01.014 29391237

[60] Liu Z Yao X Sun B Jiang W Liao C Dai X. Pretreatment with kaempferol attenuates microglia-mediate neuroinflammation by inhibiting MAPKs?NF??B signaling pathway and pyroptosis after secondary spinal cord injury. Free Radic Biol Med (2021) 168:142–54. doi: 10.1016/j.freeradbiomed.2021.03.037 33823244

[61] Cao C-Y Yang Y-X Xie Z Chen X Shi X-W Yin X. Derivatives of sarcodonin a isolated from sarcodon scabrosus reversed LPS-induced M1 polarization in microglia through MAPK/NF-kappa b pathway. Bioorganic Chem (2022) 125:105854. doi: 10.1016/j.bioorg.2022.105854 35597110

[62] Liu B Zhang Y Yang Z Liu M Zhang C Zhao Y. Omega-3 DPA protected neurons from neuroinflammation by balancing microglia M1/M2 polarizations through inhibiting NF-kappa B/MAPK p38 signaling and activating neuron-BDNF-PI3K/AKT pathways. Mar Drugs (2021) 19:587. doi: 10.3390/md19110587 34822458 PMC8619469

[63] Gorski JA Zeiler SR Tamowski S Jones KR. Brain-derived neurotrophic factor is required for the maintenance of cortical dendrites. J Neurosci (2003) 23:6856–65. doi: 10.1523/JNEUROSCI.23-17-06856.2003 PMC6740724 12890780

[64] Wu S-Y Pan B-S Tsai S-F Chiang Y-T Huang B-M Mo F-E. BDNF reverses aging-related microglial activation. J Neuroinflamm (2020) 17:210. doi: 10.1186/s12974-020-01887-1 PMC7362451 32664974

[65] Miao H Li R Han C Lu X Zhang H. Minocycline promotes posthemorrhagic neurogenesis *via* M2 microglia polarization *via* upregulation of the TrkB/BDNF pathway in rats. J Neurophysiol (2018) 120:1307–17. doi: 10.1152/jn.00234.2018 29790836

[66] Numakawa T Suzuki S Kumamaru E Adachi N Richards M Kunugi H. BDNF function and intracellular signaling in neurons. Histol Histopath (2010) 25:237–58. doi: 10.14670/HH-25.237 20017110

[67] Zhang J Yao W Hashimoto K. Brain-derived neurotrophic factor (BDNF)-TrkB signaling in inflammation-related depression and potential therapeutic targets. Curr Neuropharmacol (2016) 14:721–31. doi: 10.2174/1570159X14666160119094646 PMC5050398 26786147

[68] Sun G Miao Z Ye Y Zhao P Fan L Bao Z. Curcumin alleviates neuroinflammation, enhances hippocampal neurogenesis, and improves spatial memory after traumatic brain injury. Brain Res Bull (2020) 162:84–93. doi: 10.1016/j.brainresbull.2020.05.009 32502596

[69] Wen AY Sakamoto KM Miller LS. The role of the transcription factor CREB in immune function. J Immunol (2010) 185:6413–9. doi: 10.4049/jimmunol.1001829 PMC5519339 21084670

[70] Haque ME Akther M Jakaria M Kim I-S Azam S Choi D-K. Targeting the microglial NLRP3 inflammasome and its role in parkinson’s disease. Mov Disord (2020) 35:20–33. doi: 10.1002/mds.27874 31680318

[71] Holbrook JA Jarosz-Griffiths HH Caseley E Lara-Reyna S Poulter JA Williams-Gray CH. Neurodegenerative disease and the NLRP3 inflammasome. Front Pharmacol (2021) 12:643254. doi: 10.3389/fphar.2021.643254 33776778 PMC7987926

[72] Swanson KV Deng M Ting JP-Y. The NLRP3 inflammasome: molecular activation and regulation to therapeutics. Nat Rev Immunol (2019) 19:477–89. doi: 10.1038/s41577-019-0165-0 PMC7807242 31036962

[73] Luo Y Reis C Chen S. NLRP3 inflammasome in the pathophysiology of hemorrhagic stroke: A review. Curr Neuropharmacol (2019) 17:582–9. doi: 10.2174/1570159X17666181227170053 PMC6712291 30592254

[74] Sun L Yong Y Wei P Wang Y Li H Zhou Y. Electroacupuncture ameliorates postoperative cognitive dysfunction and associated neuroinflammation *via* NLRP3 signal inhibition in aged mice. CNS Neurosci Ther (2022) 28:390–400. doi: 10.1111/cns.13784 34951130 PMC8841296

[75] Wei P Yang F Zheng Q Tang W Li J. The potential role of the NLRP3 inflammasome activation as a link between mitochondria ROS generation and neuroinflammation in postoperative cognitive dysfunction. Front Cell Neurosci (2019) 13:73. doi: 10.3389/fncel.2019.00073 30873011 PMC6401615

[76] O’Brien WT Pham L Symons GF Monif M Shultz SR McDonald SJ. The NLRP3 inflammasome in traumatic brain injury: potential as a biomarker and therapeutic target. J Neuroinflamm (2020) 17:104. doi: 10.1186/s12974-020-01778-5 PMC7137518 32252777

[77] Hanslik KL Ulland TK. The role of microglia and the Nlrp3 inflammasome in alzheimer’s disease. Front Neurol (2020) 11:570711. doi: 10.3389/fneur.2020.570711 33071950 PMC7530640

[78] Li D Chen M Meng T Fei J. Hippocampal microglial activation triggers a neurotoxic-specific astrocyte response and mediates etomidate-induced long-term synaptic inhibition. J Neuroinflamm (2020) 17:109. doi: 10.1186/s12974-020-01799-0 PMC7140340 32264970

[79] Chen L Dong R Lu Y Zhou Y Li K Zhang Z. MicroRNA-146a protects against cognitive decline induced by surgical trauma by suppressing hippocampal neuroinflammation in mice. Brain Behav Immun (2019) 78:188–201. doi: 10.1016/j.bbi.2019.01.020 30685530

[80] Cheng L Chen Z Wang L Lan Y Zheng L Wu F. Propofol partially attenuates complete freund’s adjuvant-induced neuroinflammation through inhibition of the ERK1/2/NF-b pathway. J Cell Biochem (2019) 120:9400–8. doi: 10.1002/jcb.28215 30536812

[81] Su W Xie M Li Y Gong X Li J. Topiramate reverses physiological and behavioral alterations by postoperative cognitive dysfunction in rat model through inhibiting TNF signaling pathway. Neuromol Med (2020) 22:227–38. doi: 10.1007/s12017-019-08578-y 31758388

[82] Liu P Gao T Li T Yang Y Xu Y Xu Z. Repeated propofol exposure-induced neuronal damage and cognitive impairment in aged rats by activation of NF-kappa b pathway and NLRP3 inflammasome. Neurosci Lett (2021) 740:135461. doi: 10.1016/j.neulet.2020.135461 33115643

[83] Wang T Weng H Zhou H Yang Z Tian Z Xi B. Esketamine alleviates postoperative depression-like behavior through anti-inflammatory actions in mouse prefrontal cortex. J Affect Disord (2022) 307:97–107. doi: 10.1016/j.jad.2022.03.072 35378150

[84] Verdonk F Petit A-C Abdel-Ahad P Vinckier F Jouvion G de Maricourt P. Microglial production of quinolinic acid as a target and a biomarker of the antidepressant effect of ketamine. Brain Behav Immun (2019) 81:361–73. doi: 10.1016/j.bbi.2019.06.033 31255681

[85] Li S Luo X Hua D Wang Y Zhan G Huang N. Ketamine alleviates postoperative depression-like symptoms in susceptible mice: The role of BDNF-TrkB signaling. Front Pharmacol (2020) 10:1702. doi: 10.3389/fphar.2019.01702 32116688 PMC7016044

[86] Zhang Z Bai H Ma X Shen M Li R Qiu D. Blockade of the NLRP3/caspase-1 axis attenuates ketamine-induced hippocampus pyroptosis and cognitive impairment in neonatal rats. J Neuroinflamm (2021) 18:239. doi: 10.1186/s12974-021-02295-9 PMC8527745 34666787

[87] Li Y Shen R Wen G Ding R Du A Zhou J. Effects of ketamine on levels of inflammatory cytokines IL-6, IL-1 beta, and TNF-alpha in the hippocampus of mice following acute or chronic administration. Front Pharmacol (2017) 8:139. doi: 10.3389/fphar.2017.00139 28373844 PMC5357631

[88] Tang XL Wang X Fang G Zhao YL Yan J Zhou Z. Resveratrol ameliorates sevoflurane-induced cognitive impairment by activating the SIRT1/NF-kappa b pathway in neonatal mice. J Nutr Biochem (2021) 90:108579. doi: 10.1016/j.jnutbio.2020.108579 33388350

[89] Li Q Zhang X Li S Li W Teng Y Zhou Y. Carnosol alleviates sevoflurane-induced cognitive dysfunction by mediating NF-kappa b pathway in aged rats. Drug Dev Res (2022) 83:1342–50. doi: 10.1002/ddr.21963 35781309

[90] Li N Ma Y Li C Sun M Qi F. Dexmedetomidine alleviates sevoflurane-induced neuroinflammation and neurocognitive disorders by suppressing the P2X4R/NLRP3 pathway in aged mice. Int J Neurosci (2022) 13:1–11. doi: 10.1080/00207454.2022.2121921 36066545

[91] Ye J-S Chen L Lu Y-Y Lei S-Q Peng M Xia Z-Y. Honokiol-mediated mitophagy ameliorates postoperative cognitive impairment induced by Surgery/Sevoflurane *via* inhibiting the activation of NLRP3 inflammasome in the hippocampus. Oxid Med Cell Longev (2019) 2019:8639618. doi: 10.1155/2019/8639618 30918581 PMC6409065

[92] Chen H Chu H Jiang Q Wang C Tian Y. Irf6 participates in sevoflurane-induced perioperative neurocognitive disorder *via* modulating M2, but not M1 polarization of microglia. Brain Res Bull (2021) 177:1–11. doi: 10.1016/j.brainresbull.2021.09.012 34536519

[93] Shen X Dong Y Xu Z Wang H Miao C Soriano SG. Selective anesthesia-induced neuroinflammation in developing mouse brain and cognitive impairment. Anesthesiology (2013) 118:502–15. doi: 10.1097/ALN.0b013e3182834d77 PMC3580002 23314110

[94] Liu H Chen B Guo B Deng X Wang B Dou X. Postconditioning with sevoflurane or propofol alleviates lipopolysaccharide-induced neuroinflammation but exerts dissimilar effects on the NR2B subunit and cognition. Mol Neurobiol (2021) 58:4251–67. doi: 10.1007/s12035-021-02402-0 33970453

[95] Shi C-X Ding Y-B Yu F Jin J Li T Ma J-H. Effects of sevoflurane post-conditioning in cerebral ischemia-reperfusion injury *via* TLR4/NF-kappa b pathway in rats. Eur Rev Med Pharmacol Sci (2018) 22:1770–5. doi: 10.26355/eurrev_201803_14595 29630125

[96] Zhao S Fan Z Hu J Zhu Y Lin C Shen T. The differential effects of isoflurane and sevoflurane on neonatal mice. Sci Rep (2020) 10:19345. doi: 10.1038/s41598-020-76147-6 PMC7652873 33168900

[97] Wang Z Meng S Cao L Chen Y Zuo Z Peng S. Critical role of NLRP3-caspase-1 pathway in age-dependent isoflurane-induced microglial inflammatory response and cognitive impairment. J Neuroinflamm (2018) 15:109. doi: 10.1186/s12974-018-1137-1 PMC5904978 29665808

[98] Jiang T Xu S Shen Y Xu Y Li Y. Genistein attenuates isoflurane-induced neuroinflammation by inhibiting TLR4-mediated microglial-polarization *in vivo* and *in vitro* . J Of Inflammation Res (2021) 14:2587–600. doi: 10.2147/JJR.5304336 PMC8216758 34168482

[99] Peng L Liu S Xu J Xie W Fang X Xia T. Metformin alleviates prolonged isoflurane inhalation induced cognitive decline *via* reducing neuroinflammation in adult mice. Int Immunopharmacol (2022) 109:108903. doi: 10.1016/j.intimp.2022.108903 PMC9190296 35709590

[100] Sun M Deng B Zhao X Gao C Yang L Zhao H. Isoflurane preconditioning provides neuroprotection against stroke by regulating the expression of the TLR4 signalling pathway to alleviate microglial activation. Sci Rep (2015) 5:11445. doi: 10.1038/srep11445 PMC4471883 26086415

[101] Li L Zuo Z. Isoflurane preconditioning improves short-term and long-term neurological outcome after focal brain ischemia in adult rats. Neuroscience (2009) 164:497–506. doi: 10.1016/j.neuroscience.2009.08.011 19679170 PMC2762026

[102] Zheng S Zuo Z. Isoflurane preconditioning induces neuroprotection against ischemia *via* activation of P38 mitogen-activated protein kinases. Mol Pharmacol (2004) 65:1172–80. doi: 10.1124/mol.65.5.1172 15102945

[103] Zhang X Lv M Zhu X Tian L Li J Shao Y. Isoflurane preconditioning ameliorates electromagnetic pulse-induced neural damage by shifting microglia polarization toward anti-inflammatory phenotype *via* upregulation of SOCS1. Int Immunopharmacol (2019) 68:48–57. doi: 10.1016/j.intimp.2018.12.064 30611001

[104] Lin D Cao L Wang Z Li J Washington JM Zuo Z. Lidocaine attenuates cognitive impairment after isoflurane anesthesia in old rats. Behav Brain Res (2012) 228:319–27. doi: 10.1016/j.bbr.2011.12.010 PMC3268839 22192381

[105] Li J Zhu X Yang S Xu H Guo M Yao Y. Lidocaine attenuates cognitive impairment after isoflurane anesthesia by reducing mitochondrial damage. Neurochem Res (2019) 44:1703–14. doi: 10.1007/s11064-019-02799-0 30989480

[106] Ma L Li J Zhou J Zhang D Xiao Z Yu T. Intravenous lidocaine alleviates postherpetic neuralgia in rats *via* regulation of neuroinflammation of microglia and astrocytes. iScience (2021) 24:102108. doi: 10.1016/j.isci.2021.102108 33604528 PMC7876569

[107] Zhang Y Tao G-J Hu L Qu J Han Y Zhang G. Lidocaine alleviates morphine tolerance *via* AMPK-SOCS3-dependent neuroinflammation suppression in the spinal cord. J Neuroinflamm (2017) 14:211. doi: 10.1186/s12974-017-0983-6 PMC5667445 29096659

[108] Hou Y Xiao X Yu W Qi S. Propofol suppresses microglia inflammation by targeting TGM2/NF-kappa b signaling. J Immunol Res (2021) 2021:4754454. doi: 10.1155/2021/4754454 34485533 PMC8410446

[109] Liu J Ai P Sun Y Yang X Li C Liu Y. Propofol inhibits microglial activation *via* miR-106b/Pi3k/Akt axis. Front Cell Neurosci (2021) 15:768364. doi: 10.3389/fncel.2021.768364 34776870 PMC8581742

[110] Qin X Sun Z-Q Zhang X-W Dai X-J Mao S-S Zhang Y-M. TLR4 signaling is involved in the protective effect of propofol in BV2 microglia against OGD/reoxygenation. J Physiol Biochem (2013) 69:707–18. doi: 10.1007/s13105-013-0247-6 23512249

[111] Gui B Su M Chen J Jin L Wan R Qian Y. Neuroprotective effects of pretreatment with propofol in LPS-induced BV-2 microglia cells: Role of TLR4 and GSK-3 beta. Inflammation (2012) 35:1632–40. doi: 10.1007/s10753-012-9478-x 22588329

[112] Chang Y Lee J-J Hsieh C-Y Hsiao G Chou D-S Sheu J-R. Inhibitory effects of ketamine on lipopolysaccharide-induced microglial activation. Mediat Inflammation (2009) 2009:705379. doi: 10.1155/2009/705379 PMC2662525 19343193

[113] Shibakawa YS Sasaki Y Goshima Y Echigo N Kamiya Y Kurahashi K. Effects of ketamine and propofol on inflammatory responses of primary glial cell cultures stimulated with lipopolysaccharide. Br J Anaesth (2005) 95:803–10. doi: 10.1093/bja/aei256 16227338

[114] Penning DH Cazacu S Brodie A Jevtovic-Todorovic V Kalkanis SN Lewis M. Neuron-glia crosstalk plays a major role in the neurotoxic effects of ketamine *via* extracellular vesicles. Front Cell Dev Biol (2021) 9:691648. doi: 10.3389/fcell.2021.691648 34604212 PMC8481868

[115] Pei Z Wang S Li Q. Sevoflurane suppresses microglial M2 polarization. Neurosci Lett (2017) 655:160–5. doi: 10.1016/j.neulet.2017.07.001 28687236

[116] Zhang L Zhang J Yang L Dong Y Zhang Y Xie Z. Isoflurane and sevoflurane increase interleukin-6 levels through the nuclear factor-kappa b pathway in neuroglioma cells. Br J Anaesthesia (2013) 110:82–91. doi: 10.1093/bja/aet115 PMC3667345 23604542

[117] Wang Q Ma M Yu H Yu H Zhang S Li R. Mirtazapine prevents cell activation, inflammation, and oxidative stress against isoflurane exposure in microglia. Bioengineered (2022) 13:521–30. doi: 10.1080/21655979.2021.2009971 PMC8805817 34964706

[118] Su D Gu Y Wang Z Wang X. Lidocaine attenuates proinflammatory cytokine production induced by extracellular adenosine triphosphate in cultured rat microglia. Anesth Analg (2010) 111:768–74. doi: 10.1213/ANE.0b013e3181e9e897 20686009

[119] Yuan T Li Z Li X Yu G Wang N Yang X. Lidocaine attenuates lipopolysaccharide-induced inflammatory responses in microglia. J Surg Res (2014) 192:150–62. doi: 10.1016/j.jss.2014.05.023 24952412

[120] Walsh CT. Propofol: Milk of amnesia. Cell (2018) 175:10–3. doi: 10.1016/j.cell.2018.08.031 30217361

[121] Miller D Lewis SR Pritchard MW Schofield-Robinson OJ Shelton CL Alderson P. Intravenous versus inhalational maintenance of anaesthesia for postoperative cognitive outcomes in elderly people undergoing non-cardiac surgery. Cochrane Database Syst Rev (2018) 8:CD012317. doi: 10.1002/14651858.CD012317.pub2 30129968 PMC6513211

[122] Li W-X Luo R-Y Chen C Li X Ao J-S Liu Y. Effects of propofol, dexmedetomidine, and midazolam on postoperative cognitive dysfunction in elderly patients: a randomized controlled preliminary trial. Chin Med J (2019) 132:437–45. doi: 10.1097/CM9.0000000000000098 PMC6595716 30707179

[123] Gianferrara T Cescon E Grieco I Spalluto G Federico S. Glycogen synthase kinase 3 beta involvement in neuroinflammation and neurodegenerative diseases. Curr Med Chem (2022) 29:4631–97. doi: 10.2174/0929867329666220216113517 35170406

[124] Yu D Jiang Y Gao J Liu B Chen P. Repeated exposure to propofol potentiates neuroapoptosis and long-term behavioral deficits in neonatal rats. Neurosci Lett (2013) 534:41–6. doi: 10.1016/j.neulet.2012.12.033 23295901

[125] Chen B Deng X Wang B Liu H. Persistent neuronal apoptosis and synaptic loss induced by multiple but not single exposure of propofol contribute to long-term cognitive dysfunction in neonatal rats. J Toxicol Sci (2016) 41:627–36. doi: 10.2131/jts.41.627 27665772

[126] Gao M Rejaei D Liu H. Ketamine use in current clinical practice. Acta Pharmacol Sin (2016) 37:865–72. doi: 10.1038/aps.2016.5 PMC4933765 27018176

[127] Mion G. Ketamine infusions for sedation in ICU. Anaesth Crit Care Pain Med (2019) 38:397–8. doi: 10.1016/j.accpm.2018.11.010 30562616

[128] Hudetz JA Iqbal Z Gandhi SD Patterson KM Byrne AJ Hudetz AG. Ketamine attenuates post-operative cognitive dysfunction after cardiac surgery. Acta Anaesthesiol Scand (2009) 53:864–72. doi: 10.1111/j.1399-6576.2009.01978.x 19422355

[129] Roytblat L Talmor D Rachinsky M Greemberg L Pekar A Appelbaum A. Ketamine attenuates the interleukin-6 response after cardiopulmonary bypass. Anesth Analg (1998) 87:266–71. doi: 10.1097/00000539-199808000-00006 9706914

[130] Nagels W Demeyere R Van Hemelrijck J Vandenbussche E Gijbels K Vandermeersch E. Evaluation of the neuroprotective effects of s(+)-ketamine during open-heart surgery. Anesth Analg (2004) 98:1595–603. doi: 10.1213/01.ANE.0000117227.00820.0C 15155311

[131] Jia X Gao Z Hu H. Microglia in depression: current perspectives. Sci China-Life Sci (2021) 64:911–25. doi: 10.1007/s11427-020-1815-6 33068286

[132] Kim I-D Lee H Kim S-W Lee H-K Choi J Han P-L. Alarmin HMGB1 induces systemic and brain inflammatory exacerbation in post-stroke infection rat model. Cell Death Dis (2018) 9:426. doi: 10.1038/s41419-018-0438-8 29555931 PMC5859283

[133] McCann ME Soriano SG. Does general anesthesia affect neurodevelopment in infants and children? BMJ-British Med J (2019) 367:l6459. doi: 10.1136/bmj.l6459 31818811

[134] Huang X Ying J Yang D Fang P Wang X Zhou B. The mechanisms of sevoflurane-induced neuroinflammation. Front Aging Neurosci (2021) 13:717745. doi: 10.3389/fnagi.2021.717745 34421578 PMC8375153

[135] Geng Y Wu Q Zhang R. Effect of propofol, sevoflurane, and isoflurane on postoperative cognitive dysfunction following laparoscopic cholecystectomy in elderly patients: A randomized controlled trial. J Clin Anesth (2017) 38:165–71. doi: 10.1016/j.jclinane.2017.02.007 28372661

[136] Chen C Shen F-Y Zhao X Zhou T Xu D-J Wang Z-R. Low-dose sevoflurane promotes hippocampal neurogenesis and facilitates the development of dentate gyrus-dependent learning in neonatal rats. ASN Neuro (2015) 7:1759091415575845. doi: 10.1177/1759091415575845 PMC4720175 25873307

[137] Xu DB Lin F He HJ Ying Y. Protective effects and underlying mechanism of sevoflurane pretreatment on cerebral ischemia-reperfusion injury in mice. J Biol Regul Homeost Agents (2020) 34:1479–85. doi: 10.23812/20-253-L 32924374

[138] Xue Z Wenda L Xiaohui C Xiaoyu Y Zhibin Z Dihan L. Dose-dependent effects of sevoflurane exposure during early lifetime on apoptosis in hippocampus and neurocognitive outcomes in sprague-dawley rats. Int J physiology pathophysiol Pharmacol (2016) 8:111–9. PMC5078483 27785338

[139] Murphy KL Baxter MG. Long-term effects of neonatal single or multiple isoflurane exposures on spatial memory in rats. Front In Neurol (2013) 4:87. doi: 10.3389/fneur.2013.00087 PMC3703565 23847588

[140] Zhang B Tian M Zhen Y Yue Y Sherman J Zheng H. The effects of isoflurane and desflurane on cognitive function in humans. Anesth Analgesia (2012) 114:410–5. doi: 10.1213/ANE.0b013e31823b2602 PMC3972614 22075020

[141] Roertgen D Kloos J Fries M Grottke O Rex S Rossaint R. Comparison of early cognitive function and recovery after desflurane or sevoflurane anaesthesia in the elderly: a double-blinded randomized controlled trial. Br J Anaesthesia (2010) 104:167–74. doi: 10.1093/bja/aep369 20042477

[142] Butterworth J Hammon JW. Lidocaine for neuroprotection: More evidence of efficacy. Anesth Analg (2002) 95:1131–3. doi: 10.1097/00000539-200211000-00001 12401579

[143] Baradari AG Habibi MR Habibi V Nouraei SM. Administration of lidocaine to prevent cognitive deficit in patients undergoing coronary artery bypass grafting and valve plasty: a systematic review and meta-analysis. Expert Rev Clin Pharmacol (2017) 10:179–85. doi: 10.1080/17512433.2017.1266252 27892772

[144] Klinger RY Cooter M Berger M Podgoreanu MV Stafford-Smith M Ortel TL. Effect of intravenous lidocaine on the transcerebral inflammatory response during cardiac surgery: a randomized-controlled trial. Can J Anesth (2016) 63:1223–32. doi: 10.1007/s12630-016-0704-0 PMC7097969 27470233

[145] Mitchell SJ Merry AF Frampton C Davies E Grieve D Mills BP. Cerebral protection by lidocaine during cardiac operations: A follow-up study. Ann Thorac Surg (2009) 87:820–5. doi: 10.1016/j.athoracsur.2008.12.042 19231397

[146] Mathew JP Mackensen GB Phillips-Bute B Grocott HP Glower DD Laskowitz DT. Randomized, double-blinded, placebo controlled study of neuroprotection with lidocaine in cardiac surgery. Stroke (2009) 40:880–7. doi: 10.1161/STROKEAHA.108.531236 PMC3718309 19164788

[147] Puljak L Kojundzic SL Hogan QH Sapunar D. Lidocaine injection into the rat dorsal root ganglion causes neuroinflammation. Anesth Analg (2009) 108:1021–6. doi: 10.1213/ane.0b013e318193873e PMC2869284 19224819

[148] Rosenblat JD Carvalho AF Li M Lee Y Subramanieapillai M McIntyre RS. Oral ketamine for depression: A systematic review. J Clin Psychiatry (2019) 80:18r12475. doi: 10.4088/JCP.18r12475 30995364

